# An electronic medical record retrieval system can be used to identify missed diagnosis in patients with primary ciliary dyskinesia

**DOI:** 10.1111/joim.20034

**Published:** 2024-11-23

**Authors:** Wangji Zhou, Qiaoling Chen, Yaqi Wang, Anhui Guo, Aohua Wu, Xueqi Liu, Jinrong Dai, Shuzhen Meng, Christopher Situ, Yaping Liu, Kai‐Feng Xu, Weiguo Zhu, Xinlun Tian

**Affiliations:** ^1^ Department of Pulmonary and Critical Care Medicine State Key Laboratory of Complex Severe and Rare Diseases Peking Union Medical College Hospital Chinese Academy of Medical Sciences, Peking Union Medical College Beijing China; ^2^ Department of Primary Care and Family Medicine State Key Laboratory of Complex Severe and Rare Diseases Peking Union Medical College Hospital Chinese Academy of Medical Sciences and Peking Union Medical College Beijing China; ^3^ Department of Laboratory Medicine and Pathobiology Faculty of Medicine University of Toronto Toronto Canada; ^4^ The State Key Laboratory for Complex, Severe, and Rare Diseases, The State Key Sci‐tech Infrastructure for Translational Medicine Peking Union Medical College Hospital Beijing China

**Keywords:** electronic medical records, missed diagnosis, primary ciliary dyskinesia, rare disease

## Abstract

**Background:**

Primary ciliary dyskinesia (PCD) is a rare, genetically heterogeneous disease. Due to difficulty accessing diagnostic services and a lack of awareness of the syndrome, clinicians often fail to recognize the classic phenotype, leading to missed diagnoses.

**Methods:**

Relevant medical records were accessed through The BIG DATA QUERY AND ANALYSIS SYSTEM of Peking Union Medical College Hospital from September 1, 2012 to March 31, 2024. The search strategy included the following key terms: (bronchiectasis OR atelectasis OR recurrent cough OR recurrent expectoration OR hemoptysis) AND (sinusitis OR nasal polyps OR otitis media OR neonatal pneumonia OR neonatal respiratory distress OR ectopic pregnancy OR infertility OR artificial insemination OR assisted reproduction OR hydrocephalus OR congenital heart disease OR organ laterality defect OR right‐sided heart OR semen OR consanguineous marriage). Patients were filtered according to inclusion and exclusion criteria, and those with clinical suspicion of PCD were invited for screening, which included nasal nitric oxide and whole exome sequencing.

**Results:**

A total of 874 medical records were retrieved. After filtering based on inclusion and exclusion criteria, 65 patients with clinical suspicion of PCD were identified, 21 of whom accepted our invitation to complete PCD‐related screening. Among them, four were diagnosed with PCD, one was diagnosed with cystic fibrosis, and one was diagnosed with immunodeficiency‐21.

**Conclusions:**

This is the first study to use an electronic medical record retrieval system to identify missed diagnoses PCD. We believe that the methods used in this study can be extended to other rare diseases in the future.

## Graphical abstract

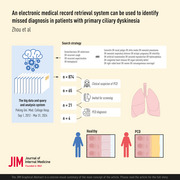

**Abbreviations**
NLP: natural language processing
nNO: nasal nitric oxide
PCD: primary ciliary dyskinesia
PUMCH: Peking Union Medical College Hospital
WES: whole exome sequencing

## Introduction

Primary ciliary dyskinesia (PCD) is a rare, genetically heterogeneous disease caused by mutations in genes that encode the specific structure or function of motile cilia. More than 40 genes have been associated with this syndrome, and additional genes continue to be discovered [1]. In most cases, PCD is transmitted by an autosomal recessive pattern of inheritance; however, X‐linked and autosomal dominant inheritance patterns are known [2, 3]. PCD is estimated to occur in approximately 1 in 20,000 live births in Europeans [4]. In China, as of May 2021, a total of 244 patients with PCD have been reported [5]. Patients with PCD typically manifest symptoms such as unexplained neonatal respiratory distress, chronic wet cough, sinusitis, otitis media, organ laterality defects, infertility, and nearly 100% concomitance of bronchiectasis in adulthood. Some patients also have congenital heart disease and hydrocephalus [1].

Early diagnosis and treatment of PCD are critically important to reduce long‐term pulmonary morbidity and prevent the development of severe bronchiectasis [6]. Correct diagnosis can also play a vital role in genetic counseling and assessing the risk of infertility. However, diagnosing PCD has historically been difficult because of the inaccessibility of diagnostic services coupled with the lack of awareness of the syndrome, leading to clinicians often failing to recognize its classic phenotype [3]. A survey of specialists in Europe reported that only a small proportion of patients estimated to have PCD have been diagnosed. Moreover, 37% of patients reported in an international survey that they had visited a doctor for PCD‐related symptoms more than 40 times before being referred for testing [7]. We speculate that patients with bronchiectasis represent a probable reservoir of undiagnosed PCD patients.

At present, electronic medical record systems have been widely popularized in large hospitals, containing extensive clinical information regarding patient phenotypes. The BIG DATA QUERY AND ANALYSIS SYSTEM of Peking Union Medical College Hospital (PUMCH) is essentially an electronic medical record retrieval system that can accurately extract structured and post‐structured field variables from electronic medical records through natural language processing (NLP). The principal stages involved in the extraction of variables from electronic medical records through the utilization of NLP technology can be broadly categorized as follows: text preprocessing, creating the extraction schema, entity recognition and relation extraction, model training, and optimization. To our knowledge, similar medical record retrieval systems exist in other large tertiary hospitals.

The first step in diagnosing rare diseases is to stratify patients into subgroups with similar phenotypic characterizations [8]. Given that PCD has a relatively specific clinical phenotype, we hypothesize that patient phenotype information obtained from electronic medical records can be utilized to identify previously unrecognized PCD patients. In this study, we used the BIG DATA QUERY AND ANALYSIS SYSTEM to search for patients with PCD‐specific phenotypes, inviting suitable candidates for PCD‐related screening such as nasal nitric oxide (nNO) measurement and whole exome sequencing (WES) to confirm our hypothesis.

## Methods

### Medical record sources and search strategies

Relevant outpatient and inpatient medical records were collected by searching the BIG DATA QUERY AND ANALYSIS SYSTEM of PUMCH from September 1, 2012 to March 31, 2024. The search strategy included the following key terms: (bronchiectasis OR atelectasis OR recurrent cough OR recurrent expectoration OR hemoptysis) AND (sinusitis OR nasal polyps OR otitis media OR neonatal pneumonia OR neonatal respiratory distress OR ectopic pregnancy OR infertility OR artificial insemination OR assisted reproduction OR hydrocephalus OR congenital heart disease OR organ laterality defect OR right‐sided heart OR semen OR consanguineous marriage).

The BIG DATA QUERY AND ANALYSIS SYSTEM is restricted to hospital personnel. Terms nested within each search box, as denoted by parentheses, are connected using the OR Boolean search operator, whereas separate search boxes are connected using the AND operator. Search boxes could be added further based on the search conditions. The search results showed detailed patient diagnosis and treatment information, including the department visited, attending physician, date of visit, medical record, diagnosis, laboratory reports, examination reports, and the patient's complete visit history.

### Inclusion and exclusion criteria

The inclusion criteria included the following: (1) recurrent cough and expectoration before the age of 18; (2) diagnosis with sinusitis or otitis media before the age of 18; (3) history of neonatal pneumonia or neonatal respiratory distress in full‐term infants; (4) organ laterality defects or right‐sided heart; and (5) family history of PCD.

The exclusion criteria were defined as follows: (1) duplicate patient entries; (2) patients lacking detailed medical record information; (3) chest CT scans indicating the absence of bronchiectasis or atelectasis; and (4) patients previously diagnosed with PCD, cystic fibrosis (CF), or immunodeficiency.

Two investigators independently reviewed the selected medical records and identified patients with clinical suspicion of PCD, according to the above criteria (meeting one or more of the inclusion criteria and not meeting all exclusion criteria). Discrepancies in patient selection between the two investigators were resolved through subsequent discussion.

### Inviting patients with clinical suspicion of PCD for screening

Patients with clinical suspicion of PCD were invited via telephone or text message to visit the Department of Pulmonary and Critical Care Medicine at PUMCH for screening. Patient demographic information (including age and gender) and medical history were collected, followed by physical examinations (with particular attention to organ laterality defects), nNO measurement, and WES. Although nNO measurement was optional, all patients underwent WES. All costs associated with WES were afforded by the researchers.

Informed consent was obtained from all patients or their legal guardians. The study was approved by the Ethics Committee of PUMCH (Identifier: JS‐2640 and I‐24PJ0537) and was carried out in accordance with the tenets of the Declaration of Helsinki.

### Diagnostic criteria

PCD was diagnosed based on at least one of the following criteria: (1) low nNO level (<77 nL/min, excluding CF) in conjunction with at least two of the four key clinical features for PCD, namely, unexplained neonatal respiratory distress in term infants, year‐round daily cough beginning before 6 months of age, year‐round daily nasal congestion beginning before 6 months of age, or organ laterality defects; (2) recognized ciliary ultrastructural defects by TEM analysis; (3) biallelic pathogenic variants in PCD‐associated genes; and (4) Kartagener syndrome [9].

If WES indicated other diseases, according to the guidelines of the American College of Medical Genetics and Genomics [10], the presence of biallelic autosomal recessive pathogenic or likely pathogenic variants, or one autosomal dominant pathogenic or likely pathogenic variant, was considered a positive result.

### Statistical analysis

Data analysis was conducted using SPSS version 22.0 software (IBM SPSS). Continuous variables are reported as mean ± standard deviation or median (interquartile range), and categorical variables are described with the corresponding percentage, No. (%). The chi‐squared test or Fisher's exact test (as appropriate) were used to compare categorical variables. *p *< 0.05 was deemed statistically significant for all analyses.

## Results

**Fig. 1:**
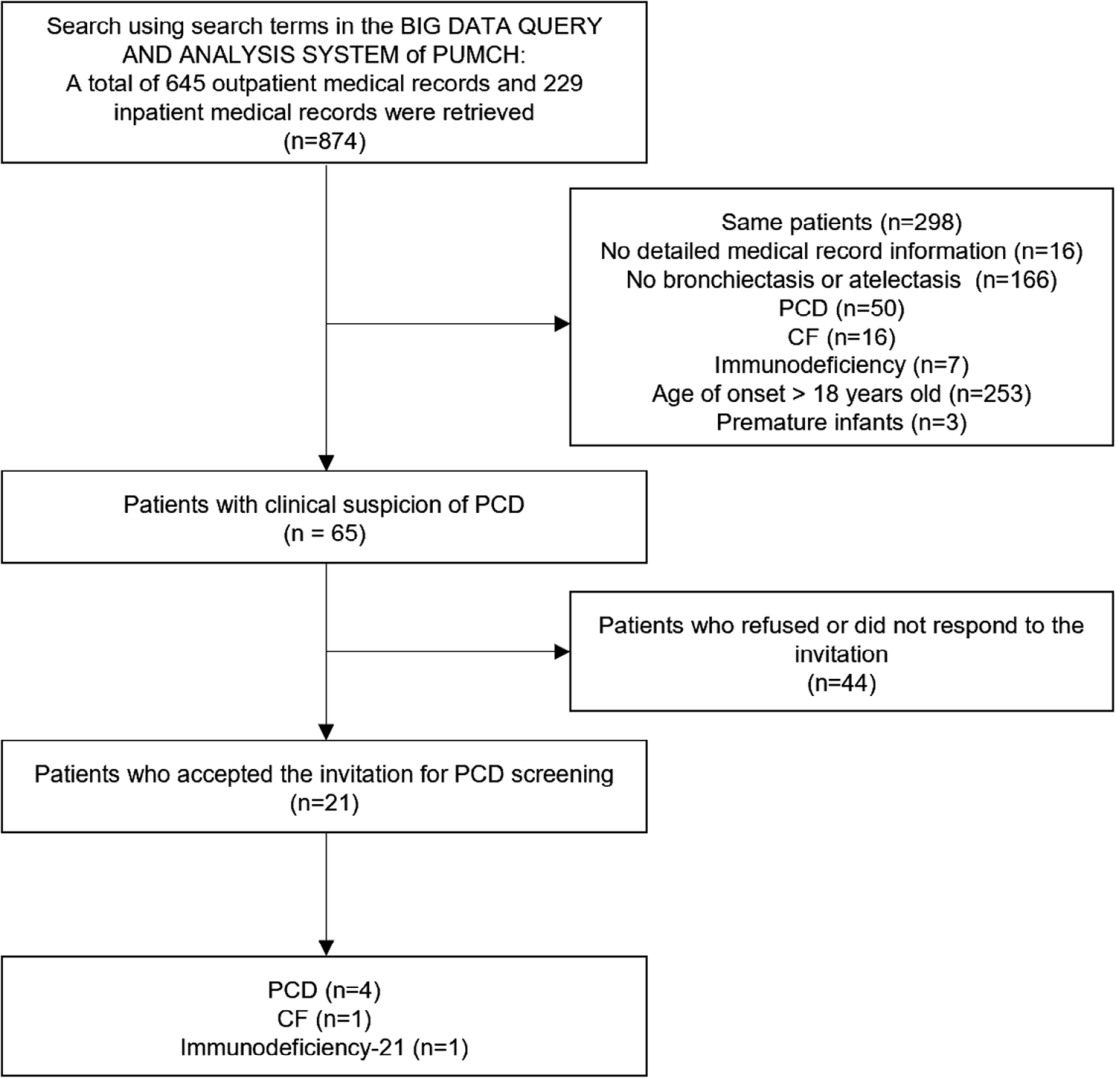
Trial inclusion and exclusion flow chart. CF, cystic fibrosis; PCD, primary ciliary dyskinesia; PUMCH, Peking Union Medical College Hospital.

A total of 874 medical records were retrieved. After filtering based on inclusion and exclusion criteria, 65 patients with clinical suspicion of PCD were identified, 21 of whom accepted our invitation to complete PCD‐related screening. Six (28.6%) patients reported positive results. Among them, four were diagnosed with PCD, one was diagnosed with CF, and one was diagnosed with immunodeficiency‐21. An overview of the study's search and inclusion process is illustrated in Fig. 1.

### Distribution of departments and doctors

Of the 65 patients with clinical suspicion of PCD, 61 (93.9%) were outpatients or inpatients of the Department of Pulmonary and Critical Care Medicine, 2 (3.1%) were outpatients of the Department of Allergy, 1 (1.5%) was an outpatient of the Department of Infectious Medicine, and 1 (1.5%) was an outpatient of the Department of Geriatrics. Twenty‐seven (41.5%) patients visited the same doctor, whereas the remaining 38 (58.5%) patients visited 21 different doctors.

All 21 patients who accepted the invitation for PCD screening were outpatients or inpatients of the Department of Pulmonary and Critical Care Medicine. A total of 12 (57.1%) patients visited the same doctor, as noted previously, whereas the remaining 9 (42.9%) patients visited 7 different doctors.

Of the six patients with positive screening results, two (33.3%) came from the same doctor's clinic yet again, whereas the remaining four (66.7%) came from the clinics of three other doctors.

There was no statistically significant difference in the distribution of departments (*p *= 0.672) and doctors (*p *= 0.388) among the three groups of patients mentioned above.

### Demographic and PCD screening results of 21 patients

Among the 21 patients who accepted the invitation for PCD screening, 15 (71.4%) were males and 6 (28.6%) were females. The average age was 29.0 ± 11.3 years old, with 16 (76.2%) adults. None of the patients exhibited organ laterality defects. Among the 17 (81.0%) patients who underwent nNO measurement, 7 (41.2%) patients had low nNO levels (<77 nL/min). Notably, this number included all four patients newly diagnosed with PCD. Additionally, all 21 patients underwent WES, with 6 (28.6%) patients reporting positive results. The four patients with PCD carry biallelic variants of *DNAAF4*, *DNAH5*, *RSPH3*, and *DNAAF1*, respectively. Moreover, one patient carries two CF‐causing *CFTR* mutations, and the other patient carries a likely pathogenic *GATA2* variant. Demographic data and detailed PCD screening results of all 21 patients are summarized in Table 1.

**Table 1:** Demographic information and primary ciliary dyskinesia (PCD) screening results of 21 patients.

				WES results				
Patient No.	Gender	Age (years)	nNO (nL/min)	Gene	Genotype	Mutations	ACMG	Diagnosis
1	Female	30	16.3	*DNAAF4*	Homozygous	c.30G>A(p.Trp10*)	LP	PCD
2	Male	15	5.8	*DNAH5*	Compound heterozygous	c.9213del(p.His3071Glnfs*5) c.6786del(p.Ser2264Valfs*12)	LP LP	PCD
3	Male	17	45.6	*RSPH3*	Compound heterozygous	c.1105C>T(p.Arg369*) c.1096C>T(p.Gln366*)	P LP	PCD
4	Female	17	11.0	*DNAAF1*	Compound heterozygous	c.376del(p.Glu126Lysfs*35) c.394del(p.Arg132Alafs*29)	P LP	PCD
5	Male	35	81.5	*CFTR*	Homozygous	c.2909G>A(p.Gly970Asp)	P	CF
6	Female	15	NA	GATA2*	Heterozygous	c.1099_1105del(p.Asp367Serfs*18)	LP	Immunodeficiency‐21
7	Female	33	81.2	*DNAH11*	Heterozygous	c.3025C>G(p.Leu1009Val)	VUS	Undefined
8	Female	20	24.4	*DNAH1*	Heterozygous	c.11726_11727delCT(p.Pro3909Argfs*33)	P	Undefined
				*DNAH11*	Compound heterozygous	c.13394C>T(p.Pro4465Leu) c.6852C>G(p.Asn2284Lys)	LPM VUS	
9	Male	20	196.7	*CFTR*	Heterozygous	c.1069G>A(p.Ala357Thr)	VUS	Undefined
10	Female	32	233.4	*CFTR*	Heterozygous	c.2991G>C(p.Leu997Phe)	VUS	Undefined
11	Female	33	101.8	Negative	/	/	/	Undefined
12	Male	39	91.4	Negative	/	/	/	Undefined
13	Female	34	NA	Negative	/	/	/	Undefined
14	Female	23	NA	Negative	/	/	/	Undefined
15	Female	42	326.4	Negative	/	/	/	Undefined
16	Female	36	47.1	Negative	/	/	/	Undefined
17	Female	32	287.4	Negative	/	/	/	Undefined
18	Male	46	193.9	Negative	/	/	/	Undefined
19	Female	55	197.6	Negative	/	/	/	Undefined
20	Female	22	66.2	Negative	/	/	/	Undefined
21	Female	14	NA	Negative	/	/	/	Undefined

*Note*: “*” Heterozygous variations in the *GATA2* gene can lead to immune dysfunction‐21, with an autosomal dominant inheritance pattern. All other discovered variations exhibit autosomal recessive inheritance.

Abbreviations: ACMG, American College of Medical Genetics and Genomics; CF, cystic fibrosis; LP, likely pathogenic; NA, not available; nNO, nasal nitric oxide measurement; P, pathogenic; VUS, variant of uncertain significance; WES, whole exome sequencing.

## Discussion

This is the first study to use an electronic medical record retrieval system (The BIG DATA QUERY AND ANALYSIS SYSTEM of PUMCH) to identify patients with missed diagnoses of PCD. We used the specific clinical phenotype of PCD as search terms in the BIG DATA QUERY AND ANALYSIS SYSTEM to identify patients with clinical suspicion of PCD and invited them for PCD‐related screening. Our results showed that among the 21 patients screened, nearly one‐third of them had a positive screening result, including 4 patients with PCD. Considering that there may still be a certain proportion of patients with PCD among the 44 patients who did not accept the invitation, our study actually underestimated the number of patients with PCD who were misdiagnosed. This confirms our hypothesis that an electronic medical record retrieval system is an effective approach for identifying previously unrecognized PCD patients.

Although there is currently no causative treatment for PCD, many studies have demonstrated the negative effect of delayed diagnosis in PCD. According to the retrospective study by Shah et al., late diagnosis of PCD is associated with decreased forced expiratory volume in 1 s (FEV_1_) and increased colonization of *Pseudomonas aeruginosa* in a large adult PCD population [6]. Recently, Gatt et al. conducted a study to determine whether early diagnosis of PCD is related to improving long‐term outcomes. They found that a later diagnosis (>8 years) was associated with lower FEV_1_ and higher rates of pulmonary exacerbations throughout childhood, although once diagnosed, there was no difference in annual decline rate [11]. In addition, several studies have been published describing the partial restoration of ciliary function in vitro using gene or transcript therapy, indicating that significant progress may be made in the treatment of PCD in the future [12]. Besides, correct diagnosis also plays a crucial role in genetic counseling and assessing infertility risk.

According to the European Respiratory Society guidelines for the diagnosis of PCD, patients with a high clinical suspicion of PCD should be recalled for further testing as new diagnostic tests become available. Patients with several of the following features: persistent wet cough, situs anomalies, congenital cardiac defects, persistent rhinitis, chronic middle ear disease with or without hearing loss, a history in term infants of neonatal upper and lower respiratory symptoms or neonatal intensive care admittance, warrant particular suspicion of PCD [4]. Consequently, we propose the hypothesis of utilizing an electronic medical record retrieval system to identify previously unrecognized PCD patients. Considering that almost 100% of patients with PCD concomitance of bronchiectasis in adulthood and that patients at PUMCH are mainly adults, we used the clinical symptoms of bronchiectasis as search terms along with other PCD‐specific symptoms, including sinusitis, otitis media, infertility, neonatal respiratory distress in term infant, organ laterality defects, congenital heart disease, or hydrocephalus. Patients usually experience these symptoms from childhood or even in the neonatal period. However, due to the difficulty in recalling these symptoms, our study limited the onset time to before adulthood.

In addition to four patients with PCD, our study also found one case of CF and one case of immunodeficiency‐21. This may be because both CF and immunodeficiency are genetic causes of bronchiectasis that can occur during adolescence. Similarly, sinusitis, otitis media, and other conditions are also observed in patients with these diseases [13, 14].

The patient's visiting department and attending physician may have impacted the results. Most of the patients in this study came from the Department of Pulmonary and Critical Care Medicine and were treated by the same doctor. Due to the use of specific PCD‐related terms in retrieving electronic medical records for this study, doctors who considered the diagnosis of PCD in their medical history inquiries were more likely to include PCD‐specific phenotype keywords in their electronic medical records. Conversely, doctors who did not consider the diagnosis of PCD were less likely to describe the specific phenotype of PCD in their medical records. For example, chest physicians were more likely to record specific phenotypes of PCD in their medical records compared to doctors from other departments, which helps explain why there were few patients outside of the Department of Pulmonary and Critical Care Medicine in this study. Nonetheless, there were no significant differences in the distribution of departments and doctors among the 65 patients with clinical suspicion of PCD, 21 patients who accepted the invitation for PCD screening, or the 6 patients with positive screening results, increasing confidence that our findings were not substantially influenced by the distribution of departments and doctors.

This study may be extended to other rare diseases in the future. Rare diseases pose a significant challenge globally, affecting approximately 10% of the population. In China, it is estimated that 49–82 million individuals have rare diseases [15]. One of the main difficulties faced by patients with rare diseases is the lack of qualified doctors specializing in these diseases, resulting in increasingly common delayed or missed diagnoses [16]. Increasing medical sub‐specialization and segmentation only serve to further exacerbate these issues. Fortunately, recent breakthroughs in big data and artificial intelligence offer powerful new methods to shorten the diagnosis time for patients. The first step in diagnosing rare diseases is to divide patients into subgroups with similar phenotypic characteristics. This approach is exemplified by the Human Phenotype Ontology, which was built to collect human phenotypic information for the differential diagnosis of rare diseases [8]. We believe that the methods used in this study can be adapted to search for specific phenotypes of other rare diseases in electronic medical record systems, ultimately helping to identify undiagnosed patients with rare diseases.

There are some limitations in this study. First, genetic causes can only be identified in approximately 70% of patients known to have PCD; therefore, negative genetic testing cannot completely exclude diagnosis [1]. Nevertheless, the combined use of transmission electron microscopy analysis and high‐speed video microscopy analysis can help improve the diagnostic rate of PCD [9]. Second, as the largest rare disease diagnosis and treatment center in China, PUMCH may have a disproportionately high number of rare disease cases, including PCD, because patients with rare diseases are referred to PUMCH from various local institutions throughout China. Third, given that this study only analyzed patients in the PUMCH, the results obtained require validation in other locations, and related studies are currently underway. Because multiple missed PCD patients were identified at PUMCH, it is reasonable to speculate that there may also be a considerable number of missed PCD patients in other large tertiary hospitals.

## Conclusions

This is the first study to use an electronic medical record retrieval system (The BIG DATA QUERY AND ANALYSIS SYSTEM of PUMCH) to screen for individuals with PCD‐specific phenotypes, thereby identifying previously undiagnosed patients with PCD. We believe that the methods used in this study can be extended to other rare diseases in the future. Ultimately, this represents a new medical paradigm beyond the traditional patient‐seeking‐doctor model, where doctors seek patients. This is of great significance in the context where specialists in rare diseases are even rarer than patients with rare diseases.

## Author contributions

Weiguo Zhu and Xinlun Tian proposed the idea and contributed a critical review of the manuscript. Wangji Zhou wrote the initial draft to which all authors contributed. Christopher Situ polished the English version of the manuscript. Wangji Zhou, Qiaoling Chen, Yaqi Wang, Anhui Guo, Aohua Wu, Xueqi Liu, Jinrong Dai, and Shuzhen Meng contributed to the collection of data. Anhui Guo assisted in the revision of the manuscript. Wangji Zhou, Yaping Liu, Kai‐Feng Xu, Weiguo Zhu, and Xinlun Tian contributed to data analysis, data interpretation, and approval of the final version. All authors read and approved the final manuscript.

## Conflict of interest statement

The authors declare that they have no conflicts of interest.

## Funding information

Chinese Academy of Medical Sciences Innovation Fund for Medical Sciences [CIFMS 2021‐I2M‐1‐056 and 2023‐I2M‐C&T‐A‐002], Capital's Funds for Health Improvement and Research [2024‐1‐2093], and National High‐Level Hospital Clinical Research Funding [2022‐PUMCH‐B‐107].

## Data Availability

Data described in this manuscript that have been collected by the research team during this study and that can be anonymized can be made available upon reasonable request to the corresponding author pending approval.
